# A novel abdominal wall entry suction device to increase Veress needle safety: A prospective cohort pilot study

**DOI:** 10.1016/j.amsu.2019.10.001

**Published:** 2019-10-07

**Authors:** Jean H.T. Daemen, Laura N. Deden, Anneline van den Ende, Milan E.J. Pijl, Cornelis H. Slump, Frits J. Berends, Edo O. Aarts

**Affiliations:** a Department of Surgery, Zuyderland Medical Centre, Heerlen, the Netherlands; b Department of Surgery, Rijnstate Hospital, Arnhem, the Netherlands; c Department of Radiology, Rijnstate Hospital, Arnhem, the Netherlands; d Department of Robotics and Mechatronics, MIRA Institute for Biomedical Technology and Technical Medicine, University of Twente, Enschede, the Netherlands

**Keywords:** Abdominal wall entry suction device, Laparoscopic surgery, Veress needle, Vascular injuries, Intestinal injuries, AA, Abdominal aorta, AWESD, Abdominal wall entry suction device, CI, Confidence interval, CT, Computed tomography, ICC, Intraclass correlation coefficient, IQR, Interquartile range, NON-AWESD, Without abdominal wall entry suction device, VC, Vena cava

## Abstract

**Background:**

In laparoscopic surgery, the Veress needle technique is most often used to initiate a pneumoperitoneum. Although low, entry-related injuries of the intestines and major vascular structures occur in 0.04–0.1% of cases. Up to 50% of these injuries remain undiagnosed at the time of surgery, resulting in mortality rates between 2.5 and 30%. In an effort to minimize such injuries we objectively assessed a novel abdominal wall entry suction device (AWESD) that was hypothesized to lift the abdominal wall and create an additional post-peritoneum safe margin for safer Veress needle introduction.

**Materials and methods:**

A prospective pilot study was conducted in which CT-scans with and without AWESD application (centered above the umbilicus) were assessed to determine its effect on the distance from the linea alba to the intestines, vena cava and abdominal aorta. Paired measurements were subjected to the Wilcoxon signed rank test.

**Results:**

Twelve participants were included. The AWESD significantly increased the median distance towards the intestines in the axial and sagittal plane (*P* = 0.01 and *P* = 0.006) from 0.93 (Inter Quartile Range (IQR): 0.33–1.51) and 0.85 (IQR: 0.32–1.47) to 1.35 (IQR: 0.39–2.27) and 1.25 (IQR: 0.42–2.10) centimeters, respectively. Similarly, for the median axial distances towards the vena cava and abdominal aorta (both *P* = 0.002) that were increased from 10.00 (IQR: 7.18–11.12) and 9.33 (IQR: 6.55–10.28) to 13.23 (IQR: 11.76–14.31) and 12.49 (IQR: 10.98–13.32) centimeters, respectively.

**Conclusion:**

The AWESD significantly increased the distances between the peritoneum and main intra-abdominal structures. However, conclusions on subsequent increased safety cannot be drawn as high-volume studies are required to determine its clinical relevance.

## Highlights

• Veress needle related injuries are rare but associated with high mortality rates.
• A novel abdominal entry suction device to increase Veress needle safety was studied.
• The device increased the distance between peritoneum and intra-abdominal structures.
• The increased distance was greatest for the vena cava and abdominal aorta.
• Despite the increased distances, the devices' clinical relevance remains unknown.

## 1 Introduction

In laparoscopy, the essential first step is to ensure abdominal access and initiate a pneumoperitoneum. This is most often established via the Veress needle primary trocar technique [[1], [2], [3], [4], [5], [6]]. This approach is particularly popular due to its simplicity, little time-consumption and effectiveness [7]. The Veress needle technique is, moreover, known to be a rather safe technique as it relies on the ability of its blunt outer sheath to retract while passing through the abdominal wall and to spring forward to cover its sharp tip when resistance diminishes. Nevertheless, at its most commonly utilized introduction site (i.e. the umbilicus), the intestines, vena cava (VC) and abdominal aorta (AA) remain at risk for significant injury. Although low, initial entry-related injuries of the intestines, vena cava and abdominal aorta occur in 1:1000–2500 of cases [[8], [9], [10], [11], [12]]. Such injuries yield, despite their low incidence, significant mortality rates of up to 2.5–30% [8,9,[11], [12], [13]]. These mortality rates are caused by the fact that 30–50% and 13–50% of intestinal and vascular injuries remain undiagnosed at the time of surgery [9,11,13]. In an effort to minimize entry-related injuries, several techniques, instruments, and approaches have been proposed, such as manual elevation of the abdominal wall or elevation utilizing clamps, an optical Veress needle [11], and many more. Recently, a novel abdominal wall entry suction device (AWESD; LapDome™, Dome Medical Technologies, New York, USA; nowadays known as LapCap_2_™, Life Care Devices, Brighton, United Kingdom) was introduced into the market of medical devices. The device was designed by its inventors to diminish entry-related injuries and offered to our hospital to be utilized in gynecological patients. The AWESD is a dome-shaped tool that was designed to lift the abdominal wall using suction and hypothesized to subsequently increase the distance towards underlying anatomical structures that are at risk during Veress needle insertion. Although, the device may already be in use by several clinics, true evidence for its working mechanism is absent. The aim of this pilot study was to objectively assess whether the AWESD is able to improve Veress needle entry safety by increasing the distance towards the intestines, AA, and VC, as compared to the situation in which no suction device is applied, utilizing Computed Tomography (CT)-scans.

## 2 Methods

### 2.1 Study design and setting

This study was a single-center prospective cohort pilot study that was conducted in the Rijnstate Hospital, Arnhem, the Netherlands. Participants were recruited between June and July 2016. CT scans were acquired prospectively, whereas patient characteristics were collected from the available medical records in a retrospective fashion. The study was approved by the local ethical board for scientific research (2016–0828) and executed fully independent of the LapCap_2_™ manufacturers. The study protocol was registered to the German Clinical Trials Register (Unique Identification Number (UIN): DRKS00017639). This report was written in compliance with the STROCSS statement [14]. All study expenses were covered by our own Research Departments of Bariatric Surgery and Radiology.

### 2.2 Participants

For this pilot study, a sample size of 12 participants was chosen [15]. Patients aged 18 years and above that were physically fit and without known abdominal pathologies, nor prior large abdominal surgeries were eligible for inclusion. Patients with a subcutaneous thickness >8 cm were excluded as their subcutis approximated the AWESD's height. In this situation it was assumed that only subcutaneous fat would be drawn into device, subsequently, losing the hypothesized peritoneal lifting power. Patients in an emergency setting, alongside pregnant or breast-feeding women were also excluded. Potentially eligible participants that required abdominal CT-examination for any reason were recruited from the Department of Radiology (Rijnstate Hospital, Arnhem, the Netherlands). Participants without a prior abdominal CT-scan or a prior scan older than 6 months were excluded because these scans served as control. However, in participants that required multiple scans (e.g. blank and arterial phase), the device could be applied during one scan, whereas the second acquisition, without device, served as control. Written informed consent was obtained from all participants upon inclusion.

### 2.3 Measurements and variables

All participants received a CT-scan according to the same imaging protocol as would have been performed without inclusion. No additional scans were acquired for AWESD assessment. Prior to scanning the AWESD was positioned on the abdominal wall and centered above the umbilicus. Moderate suction was applied (up to 60 mmHg). Following acquisition, scans were verified for sufficient quality and reconstructed. Four different measures were obtained from all AWESD CT-scans. AWESD_axial_ and AWESD_sagittal_ were defined as the distance from the anterior surface of the linea alba to the anterior intestinal wall, measured in the axial and sagittal plane, respectively. The AWESD_VC_ and AWESD_AA_ were defined as the axial distance from the anterior surface of the linea alba to the VC and AA's anterior wall, respectively. These four measures were, in like manner, derived from all scans without AWESD (i.e. NON-AWESD). The AWESD's empty space (AWESD_empty_), defined as the maximum axial distance from the device's posterior surface to the cutis layer was solely obtained from AWESD scans. All measurements were performed by the same two researchers and an experienced radiologist to assess inter-observer reliability.

### 2.4 Statistical analyses

Statistical analyses were performed using SPSS statistics (IBM Corp. Released 2017. IBM SPSS Statistics for MacOS, Version 25.0, Armonk, NY, USA). The distribution of continuous variables was assessed statistically utilizing the Shapiro-Wilk test, and assessed visually by inspection of normal probability plots and histograms. Continuous variables were denoted as mean and standard deviation (SD) or median and interquartile range (IQR) in the presence of skewness. CT scans were assessed in a pairwise manner. The mean of all observers was used to compare AWESD and NON-AWESD measurements, that were, if normally distributed, assessed using a paired samples T-test. If not, the non-parametrical Wilcoxon signed rank test was used. *P*-values ≤0.05 were considered to be statistically significant. Inter-observer reliability was assessed by the Intraclass Correlation Coefficient (ICC) and 95% confidence interval (95% CI) using a consistency, two-way random effects model based on the mean of all three raters. ICC values were interpreted as follows: values less than 0.50 reflected a poor reliability while values between 0.50 and 0.75, 0.75–0.90, or greater than 0.90 indicated moderate, good and excellent reliability, respectively [16].

### 2.5 AWESD

**Fig. 1:**
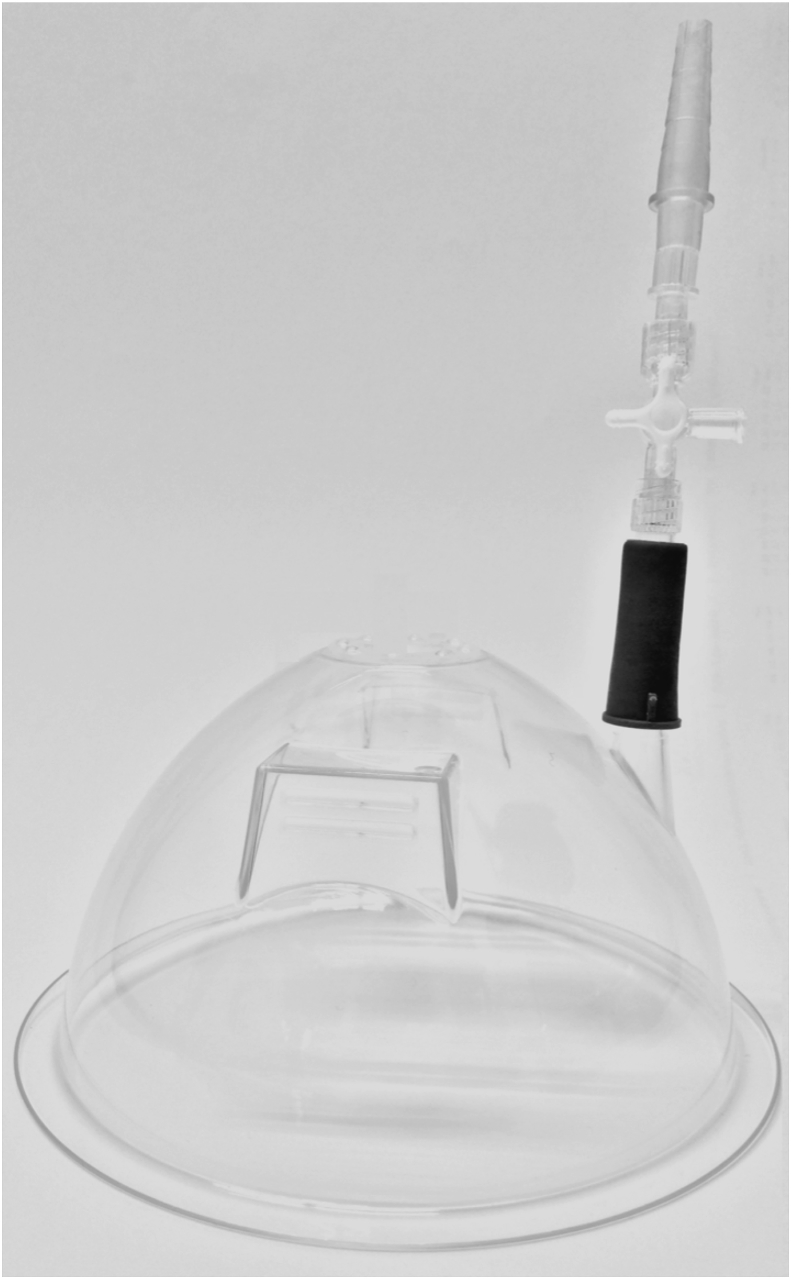
The abdominal wall entry suction device (AWESD).

The AWESD is a plastic, disposable, dome shaped device (Fig. 1) that is equipped with a central soft-plastic Veress needle insertion port on top. In addition, a suction channel was located para-centrally to induce a vacuum-like state and produce lifting power.

## 3 Results

**Fig. 2:**
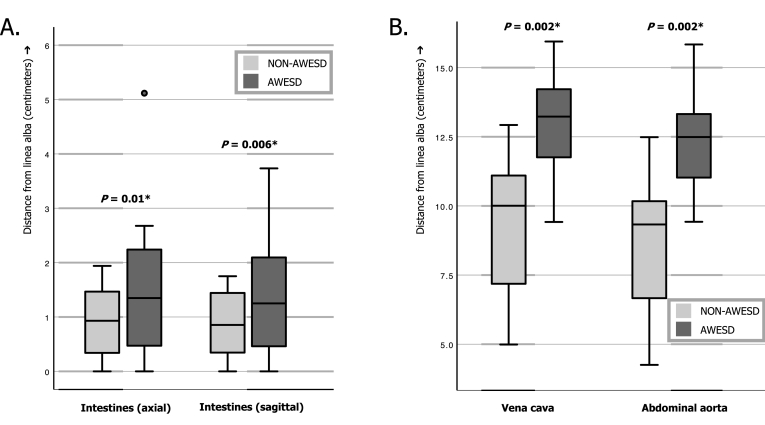
Comparison of the distances from the linea alba to the A) intestines in the axial and sagittal plane, and B) to the vena cava and abdominal aorta, without and following AWESD application. NON-AWESD: without abdominal wall entry suction device. AWESD: abdominal wall entry suction device; *: statistically significant at the 5% level.

Between the 13th of June and 21st of July 2016, twelve participants were included. Of these participants, 8 were males and 4 females with a median age of 59.5 (IQR: 51.00–72.75) years. AWESD application was successful in all participants. In one individual, application of the device caused too much pain and was removed before imaging. This participant was subsequently excluded and substituted. Although no major adverse events occurred, 3 participants demonstrated umbilical serosanguineous secretion, while all subjects demonstrated temporal skin redness underlying the AWESD. The AWESD significantly increased the median distance towards the intestines in the axial and sagittal plane (*P* = 0.01 and *P* = 0.006) from 0.93 (IQR: 0.33–1.51) and 0.85 (IQR: 0.32–1.47) to 1.35 (IQR: 0.39–2.27) and 1.25 (IQR: 0.42–2.10) centimeters, respectively (see Table 1 and Fig. 2A). An identical, significant effect was seen for the median distances towards the vena cava (*P* = 0.002) and abdominal aorta (*P* = 0.002) that were measured in the axial plane. The median distances to the vena cava and abdominal aorta were increased from 10.00 (IQR: 7.18–11.12) and 9.33 (IQR: 6.55–10.28) to 13.23 (IQR: 11.76–14.31) and 12.49 (IQR: 10.98–13.32) centimeters following AWESD application, respectively (see Table 1 and Fig. 2B).

**Table 1:** Median distances measured on AWESD and NON-AWESD scans with respect to the linea alba, based on the means of all 3 raters.

	AWESD, median (IQR)	NON-AWESD, median (IQR)	P-value
Intestines			
Axial (cm)	1.35 (0.39–2.27)	0.93 (0.33–1.51)	0.01*
Sagittal (cm)	1.25 (0.42–2.10)	0.85 (0.32–1.47)	0.006*
Vena cava (cm)	13.23 (11.76–14.31)	10.00 (7.18–11.12)	0.002*
Abdominal aorta (cm)	12.49 (10.98–13.32)	9.33 (6.55–10.28)	0.002*
Empty (cm)	2.15 (0.95–2.39)	–	–

AWESD: abdominal wall entry suction device; IQR: interquartile range; NON-AWESD: without abdominal wall entry suction device; cm: centimeters; *: statistically significant at the 5% level.

The device's median empty space (AWESD_empty_), measured in the axial plane, was 2.15 (IQR: 0.95–2.39, Table 1) centimeters. For all but one measurement the ICC was ≥0.962 with a 95% CI lower bound ≥0.901, indicating excellent reliability. For NON-AWESD_AA_, the ICC was 0.824 (95% CI: 0.535–0.945), indicating moderate to excellent reliability (see Table 2).

**Table 2:** Inter-rater reliability of all measurements performed by 3 raters.

	AWESD, ICC (95% CI)	NON-AWESD, ICC (95% CI)
Intestines		
Axial	0.993 (0.983–0.998)	0.983 (0.956–0.995)
Sagittal	0.991 (0.976–0.997)	0.980 (0.947–0.994)
Vena cava	0.983 (0.955–0.995)	0.987 (0.966–0.996)
Abdominal aorta	0.992 (0.980–0.998)	0.824 (0.535–0.945)
Empty	0.993 (0.983–0.998)	–

AWESD: abdominal wall entry suction device; ICC: intraclass correlation coefficient; CI: confidence interval; NON-AWESD: without abdominal wall entry suction device.

**Fig. 3:**
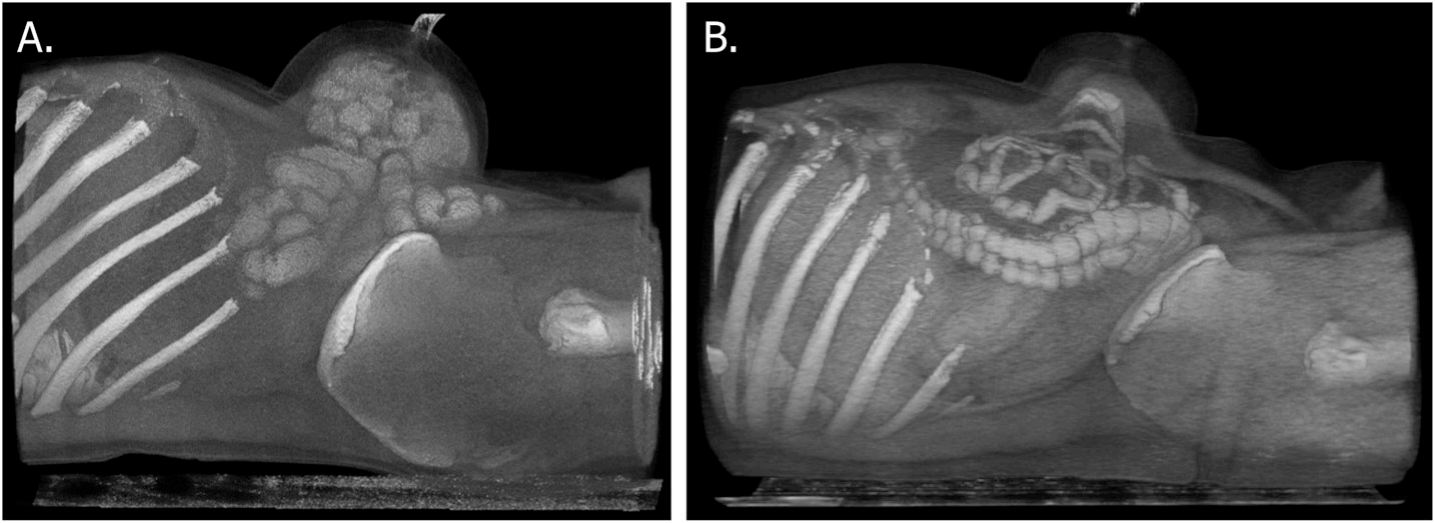
Three-dimensional reconstructions of two patients following AWESD application.

Fig. 3 A&B show examples of three-dimensional AWESD scan reconstructions for two participants in which the small intestines were drawn into the AWESD. This phenomenon was seen in 8 participants, while in the remaining 4 participants, the colon was drawn into the device.

## 4 Discussion

The aim of this prospective pilot study was to objectively assess whether the AWESD was, as intended by its inventors, able to create an additional safe zone (i.e. increased distance to the large abdominal vascular structures and intestines) behind the peritoneum to allow safer Veress needle entry. Following inter-individual assessment of CT scans, with and without AWESD application, it was found that the AWESD significantly increased the distance from the linea alba to the vena cava, abdominal aorta and intestinal wall. The effect was greatest for the vena cava and abdominal aorta with a median increase in distance of 3.23 and 3.16 cm, respectively. The AWESD may subsequently serve as a clinically valuable tool to create an additional buffer that may be able to diminish vascular injuries and subsequent death. In contrast, it may be questioned whether the significantly increased median distance towards the intestines of 4.2 and 4.0 mm yields any clinically relevant value in the prevention of entry-related injuries. This is an important aspect as intestinal injuries are more common than vascular ones. It may, moreover, be questioned whether the effects observed could also be achieved by other techniques, such as manual elevation and elevation utilizing clamps.

A randomized study by Briel et al. [17], demonstrated that manual elevation facilitated no peritoneal lift, no additional safety for Veress needle entry, nor was it easier to perform. Cakir et al. [18], assessed the effect of two towel clamps positioned on the linea alba following incision and blunt dissection, and found an increased mean distance of 11.8 mm. The difference regarding both methods can possibly be explained by the fact that the subcutaneous layer catches (i.e. compensates for) the reaction of cutaneous elevation, subsequently, preventing peritoneal lift. This could also explain the minimal increase in distances to the intestines upon AWESD application.

Entry related injuries to the intestines and vasculature may also be diminished by introduction at Palmer's point or by an open approach. Nevertheless, the point of primary introduction depends on the target organ, while an open approach, such as the Hasson-technique may be at least challenging, nor time-efficient in our ever-growing population of morbidly obese patients.

The AWESD's median empty space was 2.15 (IQR: 0.95–2.39) centimeters. However, in contrast to its intended use in an operating room setting, participants were conscious upon AWESD application and likely to exert active muscle defense. Subsequently, it was hypothesized that the distances towards the vena cava, abdominal aorta may be even further increased under general anesthesia.

As mentioned before, patients with a subcutaneous thickness >8 cm were excluded because in these patients peritoneal lifting power may be lost. Nevertheless, it is possible to apply the AWESD in obese and very thin patients by utilizing larger and smaller diameter devices. However, such AWESDs with different diameters were not available for this study.

One of the participants demonstrated a distance of zero centimeters from the linea alba to the intestinal wall. On second look, this was found to be caused by an umbilical herniation. Discretion is subsequently advised in patients with a history of abdominal or umbilical herniations. As aforementioned, the small and large intestines were, respectively, drawn into the AWESD in 8 and 4 participants. These structures, drawn into the AWESD, may consequently become more prone to perforation; particularly alarming for the colon due to its large burden of microorganisms [19].

### 4.1 Limitations

From a logical point of view; if one creates an additional post-peritoneum safe zone, by increasing the distance to the intestines and large vessels, the incidence of entry-related injuries should diminish. Nevertheless, the authors agree that a comparative study, using a control group without AWESD application to assess the potential differences in occurrence of entry-related injuries should be subject of future research. However, such a study would be limited by the large number of subjects required to achieve statistical power because of the low incidence of entry-related injuries. A randomized controlled trial including 235,458 participants (experimental and control group combined) would be required to have an 80% chance of detecting a significant difference at the 5% level, achieving a decrease in vascular complication rates from 0.04% in the control group to 0.02% in the experimental AWESD group.

Although this imaging-study was the first one in its kind that aimed to objectively assess the AWESD's intended working mechanism, the authors agree that no substantiated clinical inferences can be drawn based on this report. Although, from a logical point of view the additional safe zone is likely to reduce the incidence of entry-related vascular injuries, it should also be mentioned that any technique to lift the abdominal wall may be able to create such an increased safe zone due to the retroperitoneal location of the vena cava and abdominal aorta.

In addition, utilizing the AWESD inescapably implies additional costs and acts during surgery, where it is unknown whether these outweigh the AWESDs’ statistical benefits.

## 5 Conclusion

The AWESD is a valid and safe tool to significantly increase the distance between the peritoneum and main intra-abdominal structures that are at risk during Veress needle introduction. This effect was greatest for the vena cava and abdominal aorta, whereas the distance towards the intestines was only increased minimally. Despite these statistical benefits, its clinical relevance is unknown and questionable, and is likely to remain unstudied due to the low complication rates and enormous number of patients required.

## Declaration of competing interest

No conflicts of interest.

## Registration of research studies

Research Registration number.

DRKS00017639.

https://www.drks.de/drks_web/navigate.do?navigationId=trial.HTML&TRIAL_ID=DRKS00017639.

## Research data

Research data available.
